# Dysregulation of *FOXG1* by ring chromosome 14

**DOI:** 10.1186/s13039-015-0129-4

**Published:** 2015-04-09

**Authors:** Daniela Alosi, Laura Line Klitten, Mads Bak, Helle Hjalgrim, Rikke Steensbjerre Møller, Niels Tommerup

**Affiliations:** Department of Cellular and Molecular Medicine, Wilhelm Johannsen Centre for Functional Genome Research, University of Copenhagen, Copenhagen, Denmark; Danish Epilepsy Centre, Dianalund, Denmark; Institute of Regional Health Services Research, University of Southern Denmark, Odense, Denmark

**Keywords:** Ring chromosome 14, 14q12, Epilepsy, *FOXG1*, Intellectual disability, Dynamic mosaicism

## Abstract

In this study we performed molecular characterization of a patient with an extra ring chromosome derived from chromosome 14, with severe intellectual disability, epilepsy, cerebral paresis, tetraplegia, osteoporosis and severe thoraco-lumbal scoliosis. Array CGH analysis did not show any genomic imbalance but conventional karyotyping and FISH analysis revealed the presence of an interstitial 14q12q24.3 deletion and an extra ring chromosome derived from the deleted material. The deletion and ring chromosome breakpoints were identified at base-pair level by mate-pair and Sanger sequencing. Both breakpoints disrupted putative long non-coding RNA genes (TCONS00022561;RP11-148E17.1) of unknown function. However, the proximal breakpoint was 225 kb downstream of the forkhead box G1 gene (*FOXG1*), within the known regulatory landscape of *FOXG1*. The patient represents the first case of a r(14) arising from an interstitial excision where the phenotype is compatible with dysregulation of *FOXG1*. In turn, the phenotypic overlap between the present case, the FOXG1 syndrome and the r(14) syndrome supports that dysregulation of *FOXG1* may contribute to the classical r(14)-syndrome, likely mediated by dynamic mosaicism.

## Background

Ring chromosome 14 (r(14)) syndrome is a rare cytogenetic condition leading to a complex array of phenotypic alterations mainly characterized by growth retardation, intellectual disability, distinct facial dysmorphism with broad and flat nasal bridge, prominent forehead, broad philtrum and thin upper lip, short stature, microcephaly, scoliosis and ocular abnormalities such as abnormal retinal pigmentation, retinitis pigmentosa, strabismus, glaucoma and abnormal macula. Furthermore the patients suffer from intellectual disability, with aggressive and hyperactive behavior in some cases. Another highly characteristic finding is drug resistant epilepsy with onset during the first year of life [1-7]. Less than 80 cases have been reported in the literature and the molecular mechanisms leading to this phenotype remain to be elucidated.

Forkhead Box G1 gene (*FOXG1*) encodes a brain-expressed winged-helix transcriptional repressor, shown to be critical for forebrain development. The gene is located on 14q12 and disruption and/or dysregulation of *FOXG1* has been shown to cause the *FOXG1*-syndrome [8-14], characterized by severe postnatal microcephaly, early-onset epilepsy, brain abnormalities, severe intellectual disability, with absent or minimal language development, deficient social interactions including poor eye contact denoting a syndromic form of autism, dyskinesia with mixed features of athetosis, chorea and dystonia, epilepsy, poor sleep patterns, irritability, excessive episodes of crying and frequent gastro-oesophageal reflux [8,9,12-15]. In contrast to the r(14)-syndrome, only a mild distinct facial dysmorphism is observed in the *FOXG1*-syndrome, and only in patients with deletions [12-16]. Here we report a patient with a deleted chromosome 14 and an extra ring chromosome, where mate pair sequencing revealed a balanced excision of a ~ 50 Mb segment of chromosome 14, with truncation of the *FOXG1* regulatory landscape, and a phenotype overlapping with the r(14)-syndrome.

## Case presentation

Our patient (deceased in 2009) was an 18 year-old female patient. She was born from healthy unrelated parents after an uncomplicated pregnancy. She presented with delayed psychomotor development from approximately 2 months of age. Subsequently, severe intellectual disability (ID), cerebral paresis, tetraplegia, osteoporosis, severe thoraco-lumbal scoliosis, loss of vision, no language and no eye-contact became clinically apparent. The patient was permanently wheelchair bound. In addition, the patient was suffering from treatment-resistant epilepsy with generalized tonic-clonic seizures (GTCS), often in series, with onset at about 1 year-of-age. Despite medical treatment the patient experienced weekly seizures throughout her life. Dysmorphic facial features were not mentioned in the medical record. Computed tomography, performed at 1 year of age, showed microcephaly. Brain magnetic resonance imaging performed at 3 years of age showed a central and cortical atrophy and hypoplasia of corpus callosum.

## Methods and results

### Karyotypyng and CGH

**Figure 1:**
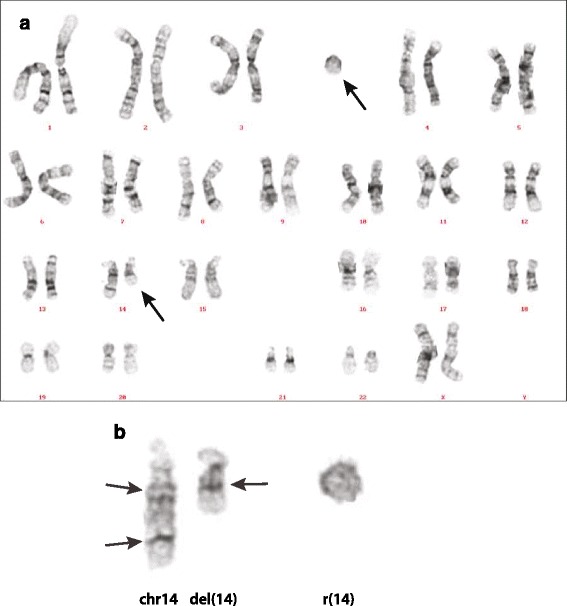
**Chromosome analysis. a**: Karyogram of the patient. **b**: Chromosome 14 and the r(14). Arrows on the left indicate the presumed position of the breakpoints. Arrow on the right side indicates the fusion of chromosome bands 14q12 and 14q24.3.

Conventional cytogenetic analysis performed during childhood showed a deletion of 14q24.2 together with a marker chromosome. The karyotype was reported as: 47,XX,del(14)(q24.2) + mar. Genome-Wide Human SNP array 6.0 (Affymetrix, Santa Clara, CA, USA) analyzed with Genotyping Console version 3.0 (GTC 3.0, Affymetrix, Santa Clara, CA, USA) did not show any unbalanced features in the genome (data not shown). Since no copy number variations were detected by array screening, the ring chromosome most likely consisted of the material missing from the derivative chromosome 14, with the reported karyotype: 47,XX, del(14)(q21q32.1) + r(14)? (Figure 1).

### Mate-pair and sanger sequencing

**Figure 2:**
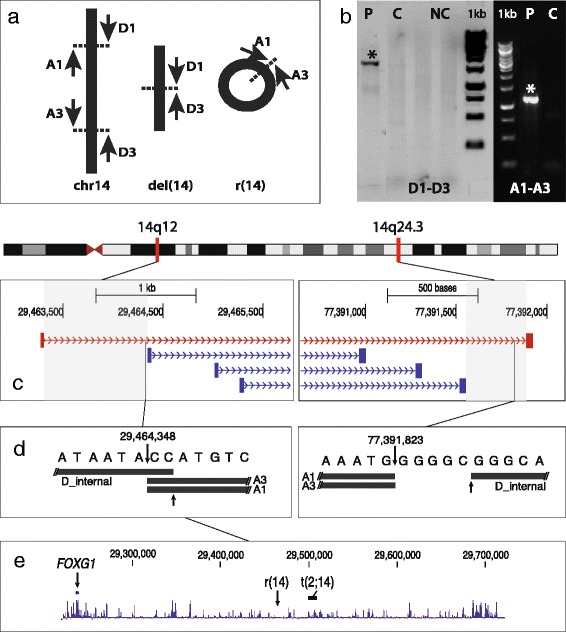
**Molecular detection of the breakpoints. a**: Schematic orientation of primers designed to amplify the breakpoints on the deleted and the ring chromosome 14. **b**: Agarose gel electrophoresis of PCR products obtained by primers D1-D3 and A1-A3. Specific amplifications (*) were observed at 62**°**C in patient (P) DNA but not in control (C) samples. NC: negative control. **c**: Mate-pair reads spanning the proximal and distal breakpoints, with resolutions corresponding to the shaded areas (proximal: ~1,000 bp; distal: ~330 bp). The thin grey lines indicate the position of the breakpoints as detected by Sanger sequencing. **d**: Position of BLAT sequences following Sanger sequencing, showing the location of the two breakpoints (upper arrows), the proximal two bp (CC) overlap and the distal 5 bp (GGGGC) deletion. **e**: Position of the proximal r(14) breakpoint in the highly conserved regulatory domain of *FOXG1*, proximal to the t(2;14)-breakpoint [13].

In order to fine map the chromosomal breakpoints, we performed Next Generation mate-pair sequencing (MPS) (Illumina Genome Analyzer II (GA-II), Illumina Inc., San Diego, CA, USA) on peripheral blood DNA. MPS libraries of patient genomic DNA were prepared according to the manufacturer’s instructions (Mate Pair Library v2, Illumina) and sequenced 2x36 bases on a Genome Analyzer IIx (Illumina). A total of 31,960,052 out of 46,169,874 read-pairs could be aligned to the Human Genome Assembly hg19 (GRCh37), using BWA [17]. Using SVDetect to search for structural variations [18], we identified four read-pairs defining the breakpoints on the r(14) chromosome and the del(14) chromosome, with a resolution of appr. 1 kb at the 14q12 breakpoint, and 332 bp at the 14q24.3 breakpoint (Figure 2c). The breakpoints were subsequently Sanger sequenced to identify their exact locations. Primers were designed with the use of the Primer3 software [19]. The PCR primer sequences were: D1: ttgatgcaaaaatcctcaataaaa (deleted derivative chromosome14, forward) and D3: gcttttcatgagctggtgtgt (deleted derivative chromosome 14, reverse). Ring specific primers were: A1: taaaacagccaaaaattgattaaa (r(14), forward) and A3: caagccatctcacattacacc (r(14), reverse) (Figure 2a). An internal sequencing primer of the D1-D3 fragment (D_internal) was: gaaggacctcttcaaggagaa (deleted derivative chromosome 14, forward). Using these primers (Figure 2a), we obtained PCR products from the patient’s DNA but not from control DNA (Figure 2b). Sequences were read on an automated capillary sequencer (ABI 3130XL, Life Technologies) and analyzed with Chromas Pro version 1.5 (Technelysium Pty Ltd).

The position of the two breakpoints were 29.464.348 (hg19, proximal breakpoint) and 77.391.823 (hg19, distal breakpoint), with a 2 bp insertion (CC) at the proximal breakpoint, and a 5 bp (GGGGC) deletion at the distal breakpoint (Figure 2d).

We did not detect any protein-coding genes in the breakpoint regions. However, both breakpoints disrupted predicted long non-coding RNA genes (lncRNA). The proximal breakpoint disrupted the lncRNA ENST00000551227.1/RP11-148E17.1. The distal breakpoint interrupted the lncRNA TCONS 00022561. Importantly, the proximal breakpoint mapped ~225 Kb downstream of *FOXG1* (Figure 2e), a member of a transcription factor family critical for cortical development [8,9,20].

### Fluorescence in Situ Hybridization (FISH)

**Figure 3:**
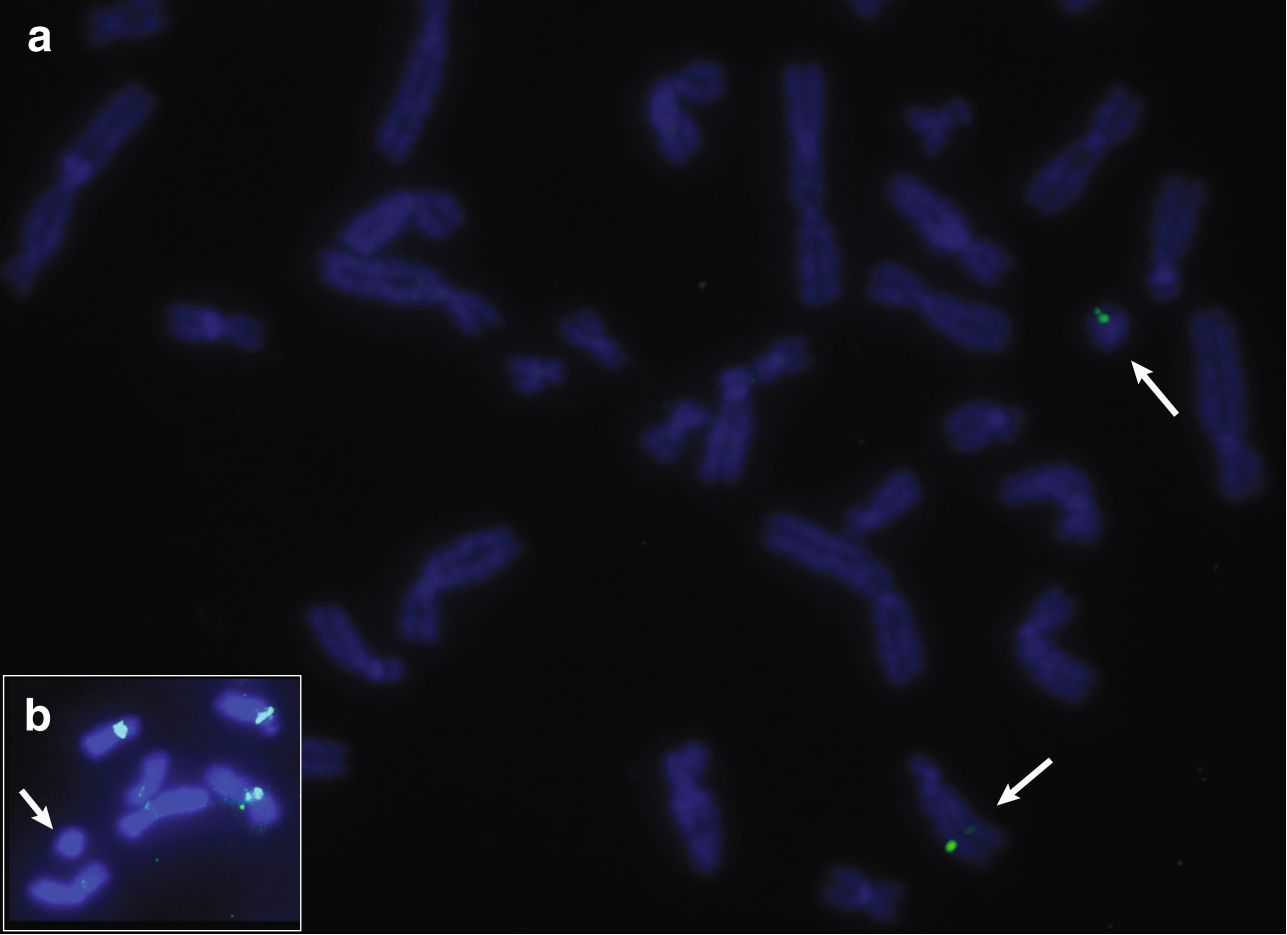
**FISH analyses. a**: The signals of BAC clone RP11-24K5 from the excised region are on the normal chromosome 14 and on the r(14). **b**: Absence of α-satellite DNA signal on the r(14).

We validated the MPS results by Fluorescence In Situ Hybridization (FISH) performed according to standard protocols using 250 ng biotin-14-dATP-labelled bacterial artificial chromosomes (BACs). We used the following BAC probes: RP11-24K5 (14q24.1), RP11-950C14 (14q24.3), RP11-35D12 (14q24.1) mapping to the predicted excised 14q24-region. The FISH analysis confirmed that the ring chromosome was composed of the excised region from 14q (Figure 3a). In order to exclude the possibility that a centromere on the ring chromosome contained centromeric chromosome 14 material, we performed FISH using α-satellite DNA probes (Oncor, Gaithersburg, MD). As expected, alpha-satellite signals were observed on all chromosomes except on the ring (Figure 3b). Since the ring was detected in all cells suggesting that it was stable, we presume that α non-satellite DNA containing neocentromere has been formed.

## Discussion

We describe a severely affected female patient with a balanced chromosomal rearrangement consisting of an interstitial excision of chromosome 14q12q24 with subsequent ring formation. The proximal breakpoint at 14q12 disrupted a predicted lncRNA gene located in a gene desert approximately 225 kb downstream of *FOXG1*. Several patients with the *FOXG1*-syndrome have been reported with deletions or balanced rearrangements in the same downstream regulatory area of *FOXG1* [e.g. 8,13]. Thus, the present breakpoint is located between *FOXG1* and the t(2;14)(q13;q13) breakpoint described in ref. 13 (Figure 2e), supporting the involvement of a long-range position effect, where distally located *cis*-regulatory elements are moved *in trans* with respect to the *FOXG1*-promoter [21]. The *FOXG1*-syndrome (former congenital variant of Rett syndrome) is a severe neurodevelopmental disorder classified by early onset of symptoms, including psychomotor delay, epilepsy and microcephaly [8-14]. These features were all present in our patient, along with many of the features of the r(14)-syndrome (Table 1). In contrast to the distinct facial features of the r(14)-syndrome, there was no report of dysmorphic features in our patient. This correlates with other reports of non-dysmorphic patients with the *FOXG1*-syndrome. Both the proximal and distal breakpoints disrupted predicted lncRNA genes. However, there is no known phenotype associated with either of these ncRNA genes, and the patient does not have any major clinical feature that has not been reported previously in the *FOXG1-* or r(14)-syndromes. It has been suggested that genes within the 14q11q13 region determine the visual impairment and epilepsy associated with the r(14)-syndrome, with *FOXG1* being one of the candidate genes [7]. Indeed, the considerable phenotypic overlap between the present case, the *FOXG1-*syndrome and the r(14)-syndrome (Table 1) suggests that dysregulation of *FOXG1* may be a significant factor behind the classical r(14)-syndrome.

**Table 1:** **Clinical features present in our patient, in the** ***FOXG1-*** **syndrome and in the r(14)-syndrome**

	**This study**	***FOXG1-*** syndrome*	r(14)-syndrome**
***Karyotype/molecular characteristics***	47, XX, del(14)(q12q24.3), + (r14)	*FOXG1* point mutations, 14q12 CNV	Ring chromosome 14
***Number of cases reported so far***	Unique	~ 50	Less than 80 cases
***Clinical features***			
***Pregnancy and neonatal period***	Normal	Normal	Normal
***Postnatal growth deficiency***		Moderate	Mild to moderate
***Intellectual disability***	Severe	Severe	Mild to severe
***Developmental delay***	Delayed psychomotor development since 2 months of age	Severe	Severe
***Postnatal microcephaly***	Yes	Postnatal microcephaly (-4/-6 SD)	Reported in the majority
***Speech-language development***	No speech	Absent or minimal speech	Severely affected
***Social interaction***	No eye-contact	Poor eye contact, denoting a syndromic form of autism	Autistic traits
***Walking***	Unable	Unable	N/A
***Loss of psychomotor skills***		Hand skills, speech, communication skills	N/A
***Skeletal abnormalities***	Osteoporosis and thoraco-lumbal scoliosis	Scoliosis, kyphosis	Scoliosis
***Facial dysmorphism***	N/A	Only in patients with deletions	Mild. Peculiar facial appearance only in patients with deletion size greater than 0.65 Mb, including blepharophimo-sis, short bulbous nose, long philtrum, epicantal folds, small mouth
***Behaviour***	N/A	Poor sleep pattern, irritability (especially in infancy), excessive crying/weeping, inappropriate laughing	Hyperactivity, aggressiveness
***Epilepsy***	Therapy-resistant epilepsy. Seizure type: GTCS, often in series. Onset: 1 year-of-age.	Infantile spasms in association with duplications, CPS, GTCS, myoclonic seizures	Early-onset (in most cases within the first year of life), typically drug-resistant GTCS, CPS
***Ocular abnormalities***	Loss of vision	Strabismus	Retinal pigmentary anomalies, retinitis pigmentosa, cataract, strabismus, maculopathy, glaucoma, myopia
***Motor and dyskinesias***	Tetraplegia	Hypotonia, spasticity, abnormal locomotion, stereotypic movements (especially hand movements), dyskinesias (chorea/athetosis/dystonia), bruxism, drooling (sialorrhea), tongue protruding movements	Hypotonia, motoric stereotypies, hand flapping, echolalia
***Gastrointestinal and respiratory systems***	N/A	Feeding difficulties, aspiration, gastro-esophageal reflux, constipation, breathing abnormalities	Susceptibility to infections of the respiratory tract
***Brain Imaging***	MRI (1994): central and cortical atrophy, corpus callosum agenesis. Suspicion of changes in cortical-spinal tracts	Simplified gyral pattern, white matter hypoplasia (frontal), hypogenesis of corpus callosum, variable mild frontal pachygyria	White matter hypoplasia, corpus callosum abnormalities, hippocampal dysmorphisms, cerebellar structural abnormalities

*FOXG1 data collected from [10;13;14;16]. **r(14) data collected from [2;3;6;7;15].

## Conclusions

We present a balanced rearrangement with an excision of 14q12q24, and an extra ring chromosome composed of the excised material. The severe clinical features are compatible with the *FOXG1-*syndrome, in line with the localization of the 14q12-breakpoint 225 kb downstream of *FOXG1,* within the regulatory domain where breakpoints are known to dysregulate*FOXG1* by long range position effects*.* Moreover, the clinical overlap between our case, the *FOXG1*-syndrome and the classical r(14)-syndrome, supports that dysregulation of *FOXG1* may be an important factor involved in the clinical features of the classical r(14)-syndrome. Since classical r(14) chromosomes would rarely have breakpoints within the *FOXG1*-regulatory domain, a likely mechanism for such a dysregulation may be the dynamic mosaicism associated with ring chromosomes, where sister chromatid exchanges within the ring lead to interlocked rings with subsequent breakage/reunion and/or aneuploidy mosaicism. Such a mechanism could also be relevant for the clinical features associated with other ring chromosomes, e.g. the development of tumors associated with the loss of specific tumor suppressors [22], and the epilepsy associated with loss of e.g. *CHRNA4* and/or *KCNQ2* in ring chromosome 20 mosaicism [23].

## Consent

The study was approved by the Ethics Committee of Western Sealand (SJ-91), and written informed consent was obtained from the patient’s parents.

## Abbreviations

r (14): Ring chromosome 14
FOXG1: Forkhead box G1
ID: Intellectual disability
CPS: Complex partial seizures
GTCS: Tonic-clonic seizures
SNP: Single nucleotide polymorphism
MPS: Mate-pair sequencing
lncRNA: Long non-coding RNA
BACs: Bacterial artificial chromosomes
